# Frequency-Reconfigurable Millimeter-Wave Rectangular Dielectric Resonator Antenna

**DOI:** 10.3390/s24123906

**Published:** 2024-06-17

**Authors:** Akrem Soltan, Rawad Asfour, Salam K. Khamas

**Affiliations:** Communications Research Group, Department of Electronic and Electrical Engineering, University of Sheffield, Mappin Steet, Sheffield S1 3JD, UK; aasoltan1@sheffield.ac.uk (A.S.); s.khamas@sheffield.ac.uk (S.K.K.)

**Keywords:** dielectric resonator antenna, millimeter-wave, PIN diode, reconfigurable antenna

## Abstract

This paper introduces an innovative and cost-effective approach for developing a millimeter-wave (mmWave) frequency-reconfigurable dielectric resonator antenna (DRA), which has not been reported before. The antenna integrates two rectangular DRA elements, where each DRA is centrally fed via a slot. A strategically positioned PIN diode is employed to exert control over performance by modulating the ON–OFF states of the diode, thereby simplifying the design process and reducing losses. In the OFF state, the first DRA, RDRA-I, exclusively supports the TE_311_ resonance mode at 24.3 GHz, offering a 2.66% impedance bandwidth and achieving a maximum broadside gain of 9.2 dBi. Conversely, in the ON state, RDRA-I and RDRA-II concurrently operate in the TE_513_ resonance mode at 29.3 GHz, providing a 2.7% impedance bandwidth and yielding a high gain of up to 11.8 dBi. Experimental results substantiate that the proposed antenna presents an attractive solution for applications necessitating frequency-reconfigurable and high-performance mmWave antennas in 5G and Beyond 5G (B5G) communication systems.

## 1. Introduction

The landscape of wireless technologies is in a perpetual state of evolution, marked by a burgeoning demand for antennas that exhibit both adaptability and a high degree of flexibility. This dynamic has spurred extensive exploration within the realm of reconfigurable antennas, where the emphasis lies in reshaping effective antenna structures through the strategic utilization of RF-lumped elements, the manipulation of substrate properties via tunable materials [1,2,3], and the integration of metamaterial-based architectures [4]. Concurrently, dielectric resonator antennas (DRAs) have emerged as a focal point of research interest owing to their intrinsic advantages, which include heightened radiation efficiency, expanded bandwidth, facile excitation, and compact form factors. Moreover, the absence of ohmic losses renders DRAs particularly appealing for applications in millimeter-wave (mmWave) and terahertz (THz) systems [5,6,7]. Consequently, many studies have explored the development of frequency, pattern, and polarization reconfigurable DRAs, as reported in [8,9,10,11,12,13,14], for example.

This convergence of dielectric resonator technology with the paradigm of reconfigurability represents a pivotal domain of inquiry, offering tantalizing prospects for augmenting antenna adaptability and efficacy across a spectrum of communication scenarios. However, published studies on mmWave reconfigurable DRA are scarce, since several mechanisms utilized to achieve DRA reconfigurability at lower frequencies could be impractical at the mmWave frequencies due to the smaller device dimensions. To the best of the authors’ knowledge, only one study is available in the open literature with a measured prototype of a pattern-reconfigurable 60 GHz DRA [15]. However, a few simulation-based studies have been reported on mmWave reconfigurable DRAs [16,17,18,19]. Therefore, a study with a measured frequency-reconfigurable mmWave DRA prototype has yet to be reported. 

This paper unveils the findings from an in-depth investigation encompassing simulated and measured novel millimeter-wave (mmWave) frequency-reconfigurable DRA architecture. The configuration features two parallel rectangular DRAs of identical dimensions. Each DRA is meticulously fed through a centrally located slot etched within a ground plane, positioned atop a dielectric substrate, with a 50 Ω feeding microstrip line integrated on the underside. The crux of achieving frequency reconfigurability lies in strategically deploying a single PIN diode switch. However, the conventional placement of these PIN diodes within the feeding slot can engender deleterious effects on antenna performance. This complication manifests during the assembly and bonding stages, potentially leading to the formation of undesired air gaps between the DRA and the ground plane.

A pioneering approach is adopted to circumvent these challenges by relocating the PIN diodes away from the feeding slots and meticulously positioning them along the microstrip feeding line. This arrangement obviates the necessity for soldering the PIN diodes [14] directly onto the feeding slot, thereby eliminating the need to carve out grooves within the physically constrained confines of the mmWave DRA. Moreover, this streamlined setup featuring a solitary switch not only simplifies the overall design, but also mitigates the occurrence of further losses. The antenna has been designed and evaluated using CST Microwave Studio. Subsequently, a prototype was fabricated and measured, demonstrating a remarkable concordance between the experimentally obtained results and those predicted through simulation.

This paper follows a structured format comprising three main sections. Section 2 introduces the proposed DRA configuration, elucidating its design and operational framework. Section 3 delves into the measured results obtained from prototypes of the DRA configuration, providing a detailed analysis of the experimental data. Finally, Section 4 offers concluding remarks and summaries, encapsulating the key insights from the study and highlighting their significance in the broader context of antenna research and development.

## 2. Antenna Configuration

**Figure 1:**
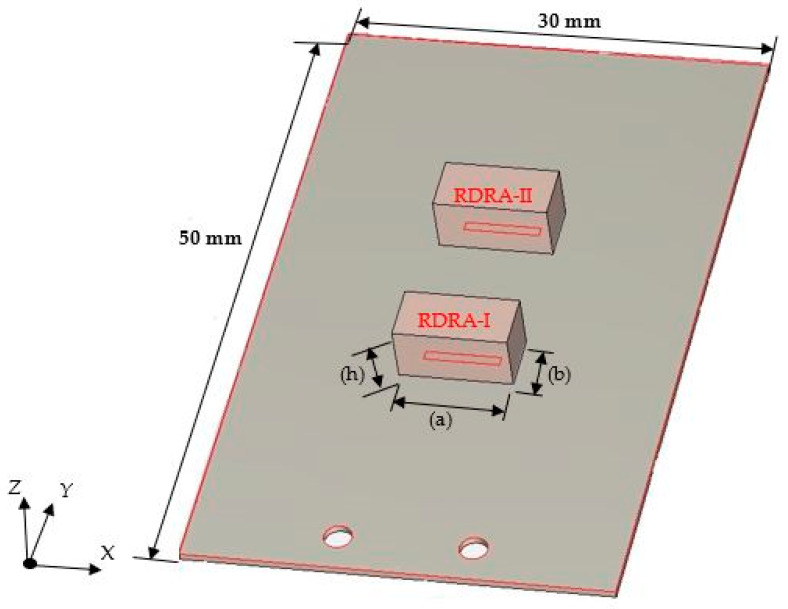
The geometry of the frequency-reconfigurable DRAs.

The DRA dimensions have been chosen to provide a configuration with a low profile and a larger rectangular footprint to minimize error during manual assembly and bonding to the ground plane. On the other hand, the DRA bulk is supported by the ground plane, which is not possible with a DRA with a higher profile and smaller footprint, which is fragile and needs extra physical support. Therefore, the DRA width, *a*, length, *b*, and height, *h*, have been chosen as *a* = 2*b* = 2*h*, as illustrated in Figure 1. These dimensions are meticulously determined through the Dielectric Waveguide Model (DWM) [20], i.e.,

(1) $$k_{y}=\frac{n\pi }{b},\;k_{z}=\frac{l\pi }{2d}$$

(2) $$k_{x}^{2}+k_{y}^{2}+k_{z}^{2}=\varepsilon_{r}k_{0}^{2}$$

(3) $$k_{x}\tan\left(\frac{k_{x}a}{2}\right)=\sqrt{\left(\left(\varepsilon_{r}-1\right)k_{0}^{2}-k_{x}^{2}\right)}$$

where $k_{x},k_{y},$ and $k_{z}$ are the wavenumbers in the *x*, *y*, and *z* directions, respectively. Substituting (1) and (2) into (3) gives a transcendental equation that needs to be solved numerically to determine the resonance frequency for the given DRA parameters of *a* = 2*b* = 2*h* and ε_r_ = 10. A MATLAB code was developed to solve (3), from which it was concluded that choosing *a* = 8 mm gives the resonance mode frequencies listed in Table 1. The DRA supports higher-order resonance modes over a 20–30 GHz frequency range. Such modes offer the needed high gains for mmWave communication. Therefore, the broadside higher-order mode of TE_311_ has been chosen as the operating mode or a single DRA. Furthermore, the DRA has been centrally fed using an aperture slot etched in the ground plane. The slot functions as a magnetic current, which runs in parallel with the aperture, to excite the magnetic field of the desired DRA mode. The slot’s length can be adjusted to maximize the energy coupling between the microturbine and the DRA. It should be noted that only the transverse electric, TE*_mnl_*, modes are excited for a rectangular DRA above a ground plane.

**Figure 2:**
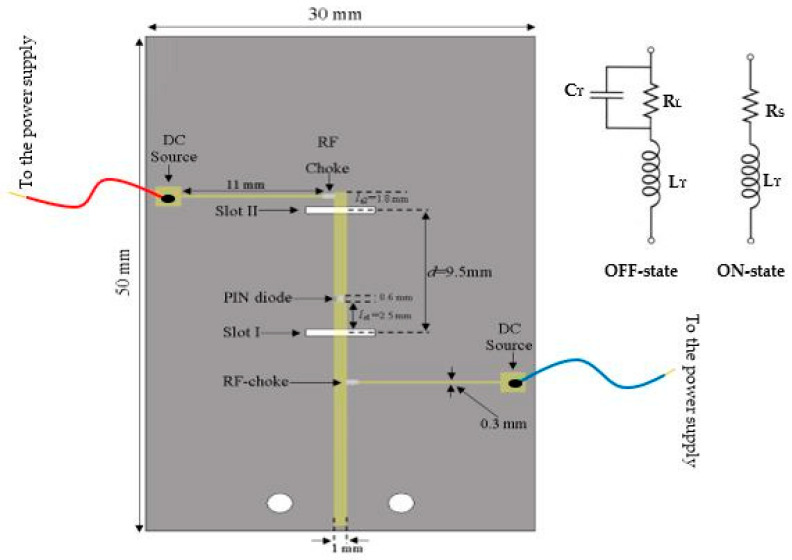
Geometry of feed network and biasing lines.

Figure 2 depicts the proposed configuration of the millimeter-wave frequency-reconfigurable rectangular DRA. During fabrication, careful consideration was given to placing the DRA on the ground plane to facilitate straightforward assembly.

Achieving frequency reconfigurability is realized by employing two parallel DRA elements, each centrally fed through a slot measuring 4 mm in length and 0.7 mm in width. These slots are intricately etched into a metal ground plane atop a Rogers RO4003C substrate, characterized by dimensions of 30 mm × 50 mm × 0.5 mm and a dielectric constant of 3.38, with a loss tangent of 0.0027. A PIN diode strategically positioned along the feeding microstrip line between the two DRAs is the crucial element enabling frequency reconfigurability. This placement ensures isolation from the DRAs, obviating the need for a physical connection between the diode and the feeding slots. Moreover, this configuration simplifies frequency tuning by utilizing only a single PIN diode instead of two with one allocated for each slot.

### 2.1. PIN Diodes and DC Biasing 

The design implementation involved the utilization of an MA4AGFCP910 PIN diode, engineered to operate effectively up to a frequency of 30 GHz. In its activated state, the diode’s electrical profile comprises a series resistance of R_S_ = 5.2 Ω and inductance with a maximum value of L_T_ = 0.5 nH. Conversely, when in the deactivated state, the diode’s equivalent circuit manifests as a C_T_ = 0.02 pF capacitor parallel to a resistance of R_L_ = 20 kΩ [21]. To bias the PIN diode, DC wires are soldered and connected to a power supply. The forward voltage is set to 1.45 V, and the current is adjusted to 10 mA. This configuration ensures that the PIN diode operates correctly, allowing it to switch between its ON and OFF states effectively. To facilitate precise simulation and analysis of the circuit’s behavior, the Touchstone lumped element within CST was leveraged to import a file encapsulating the pertinent parameters for both the activated and deactivated states of the PIN diode’s equivalent circuit. This method allows for the seamless integration of the diode’s characteristics into the simulation environment, enabling accurate assessment based on the specific values of the passive elements. Moreover, this approach streamlines the process of updating the values of these elements, eliminating the need for manual input into the simulation software.

Figure 2 illustrates the feed and biasing network, showcasing the positioning of the PIN diode between the two DRAs at the terminus of the first stub. Biasing of the PIN diode was accomplished by interfacing a DC power source with the feeding microstrip line via two thinner microstrip lines, each measuring 11 mm in length and 0.3 mm in width. These biasing microstrip lines were strategically placed at the substrate’s lower side’s upper left- and lower right-hand sides. Additionally, the incorporation of RF chokes, realized through two 30 nH inductors, effectively blocks any propagation of mmWave current along the microstrip biasing lines. Each of the two biasing lines connects at one end to the DC power supply and at the other to an RF choke. 

### 2.2. Distance between the DRAs 

**Figure 3:**
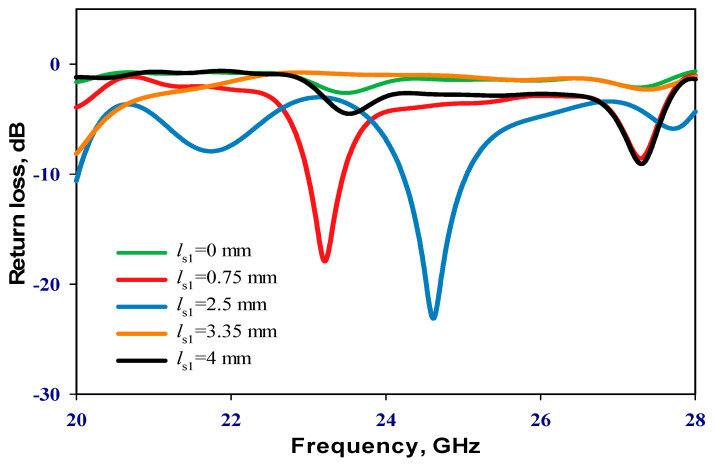
Reflection coefficient of RDRA-I with different stub lengths when the PIN diode is OFF.

**Figure 4:**
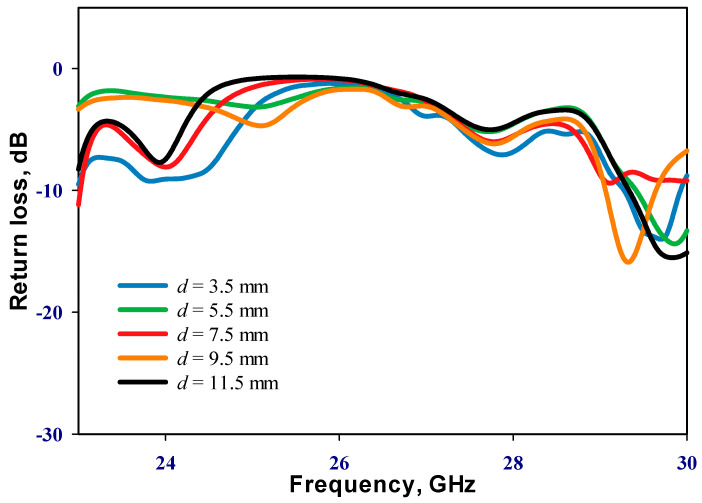
Reflection coefficient as a function of the separation distance (*d*) between the two rectangular DRAs.

**Figure 5:**
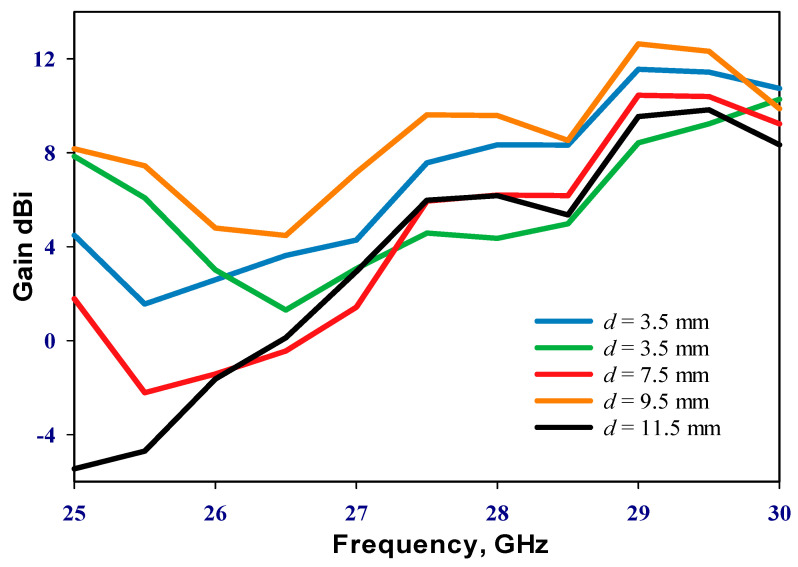
Realized gain as a function of the separation distance (*d*) between the two rectangular DRAs.

**Figure 6:**
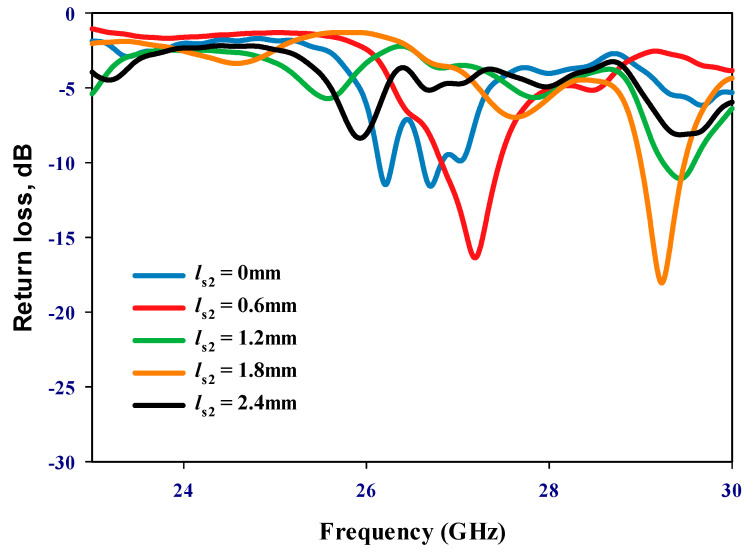
Reflection coefficient when the PIN diode is ON.

Emphasizing the significance of the distance between the two DRAs, it is imperative to note that during the inactive state of the PIN diode, only RDRA-I receives input power, with RDRA-II remaining parasitic. Conversely, upon transition to the active state, both DRAs engage, inducing a shift in operational frequency. An initial parametric analysis was conducted under the PIN diode’s inactive state, with RDRA-II excluded, as illustrated in Figure 3. Optimal matching occurs at a stub length (*l_s_*_1_) of 2.5 mm, resonating at 24.3 GHz. In the finalized prototype, both DRAs are operational, with the PIN diodes governing frequency modulation between active and inactive states. Consequently, determining the optimal distance, *d*, between the centers of the two DRAs becomes crucial. The proposed configuration was simulated by incrementally increasing *d* from 3.5 mm to 11.5 mm in 2 mm intervals when the PIN diode is ON. This systematic exploration aims to identify the most effective arrangement for the reconfigurable prototype. The investigation of the reflection coefficient, as depicted in Figure 4, revealed that an impedance bandwidth of approximately 2.7% within the frequency span of 29–29.7 GHz can be achieved when d is set to 9.5 mm, corresponding to *d* = 0.95$\lambda_{0}$ at 30 GHz. The antenna’s reflection coefficient shows the diverse resonant lengths of ls_2_. Further insights into the performance are provided by examining the variation in broadside realized gain as a function of *d*, as shown in Figure 5. Notably, the maximum gain of 12.5 dBi is attained at 29.3 GHz when *d* is configured at 9.5 mm. In addition, Figure 6 delves into the impact of varying the length of stub *l_s2_*, ranging from 0 to 2.4 mm for the second DRA. After careful evaluation, the ideal stub length for the second DRA is 1.8 mm. This specific length correlates with a higher gain than other lengths and leads to the excitation of the TE_513_ resonance mode at 29.3 GHz, resulting in a gain of 12.5 dBi. This meticulous optimization ensures that the antenna operates at its peak performance with desirable characteristics.

### 2.3. Performance of a Single DRA

**Figure 7:**
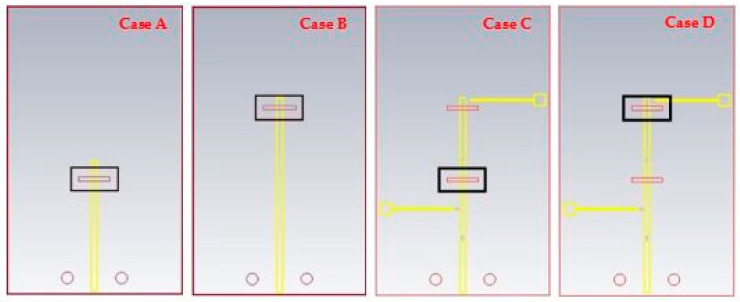
The geometry of the proposed antenna with varying presence of the PIN diode and DRAs.

Figure 7 presents four cases of the proposed antenna geometry with varying presence of the PIN diode, slot, and DRAs. In Case A, the geometry illustrates the absence of RDRA-II and its feeding slot, showcasing how the antenna performs under these conditions. Similarly, Case B demonstrates the absence of RDRA-I and its feeding slot. Case C, on the other hand, shows the absence of RDRA-II, but includes both its feeding slot and the PIN diode, highlighting how these additional elements influence the antenna’s performance. Finally, Case D presents the absence of RDRA-I with its feeding slot and PIN diode present, offering further insight into the antenna’s behavior in these configurations.

**Figure 8:**
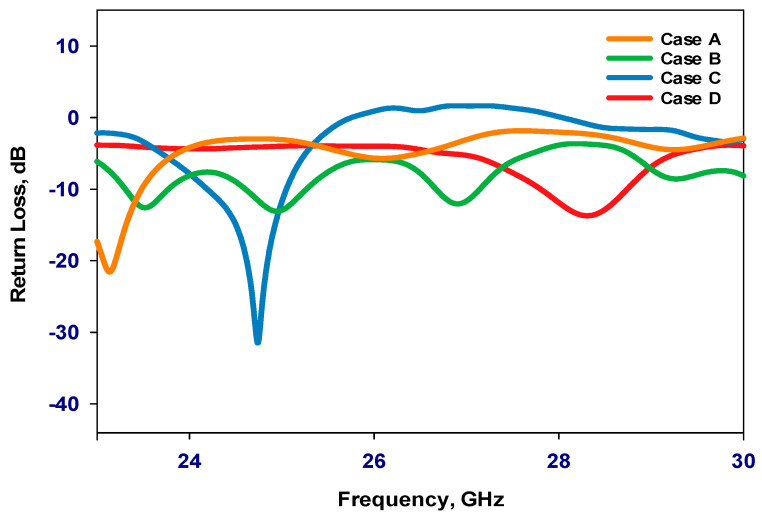
Reflection coefficient with varying presence of the PIN diode and DRAs.

Figure 8 illustrates the reflection coefficient for all cases. From these results, it can be noted that in Case A, the antenna primarily relies on the excitation of the isolated RDRA-I, resulting in an operational frequency of 23.2 GHz. In Case B, the antenna operation depends on the excitation of RDRA-II, yielding narrow bandwidths with operational frequencies at 25 GHz and 27 GHz. In Case C, the antenna again relies on the excitation of RDRA-I, achieving an operational frequency of 24.7 GHz and a gain of 6.5 dBi. The absence of RDRA-II, in this case, alters the antenna’s impedance characteristics and radiation properties, highlighting RDRA-II’s influence on the overall performance. Conversely, in Case D, the antenna configuration shifts to the excitation of RDRA-II in the absence of RDRA-I, leading to an operational frequency of 28.3 GHz and a gain of 8.2 dBi. However, the optimum performance is achieved when the two DRAs exist simultaneously with the PIN diode in between to achieve reconfigurability and enhanced gain.

The input power at the port of the proposed antenna is set to 0.5 W. The maximum emitting power depends on the state of the PIN diode. When the PIN diode is ON, the antenna achieves a maximum emitting power of 0.442 W (−3.55 dBW). Conversely, when the PIN diode is OFF, the maximum emitting power slightly decreases to 0.438 W (−3.58 dBW).

## 3. Results

**Figure 9:**
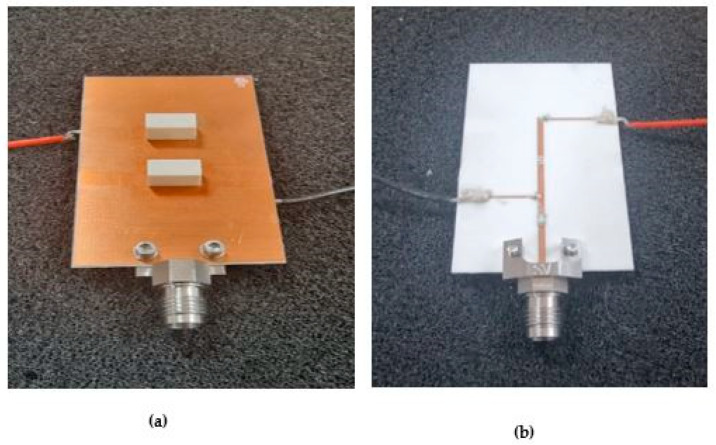
(**a**) Assembled antenna. (**b**) Bottom view of the feed network.

T-Ceramic fabricated a prototype of the antenna using Alumina [22]. The DRA is placed on top of a ground plane with an outlined DRA position using silkscreen ink to minimize measurement errors due to DRA–feed misalignment. Bonding between the DRA and the ground plane is achieved using short strips of fragile double-sided adhesive copper tape with a thickness of 0.036 mm. Figure 9 illustrates both the top and bottom views of the assembled antenna and feed network. The feed network was fabricated at the Wrekin-circuits workshop [23]. The dimensions of the PIN diode are 0.6 mm × 0.36 mm × 0.19 mm, which are too small for handling or soldering. Therefore, silver paste is used to attach the PIN diode ends to the feeding microstrip line. The |S11| was measured using an E5071C vector network analyzer connected to a 50 Ω coaxial cable through a 2.92 mm SMA connector. The equipment was calibrated using Agilent’s 85052D calibration kit. The radiation patterns were measured using the SNF-FIX-1.0 Spherical mmWave Measurement System [24].

### 3.1. OFF State Scenario

**Figure 10:**
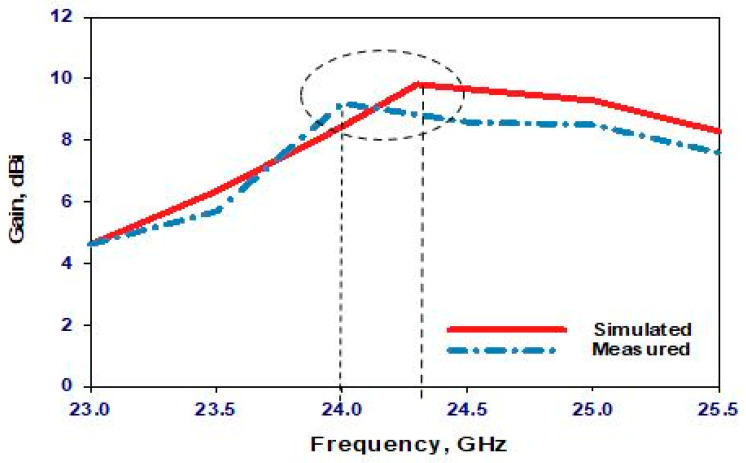
Reflection coefficient and VSWR when the PIN diode is OFF.

**Figure 11:**
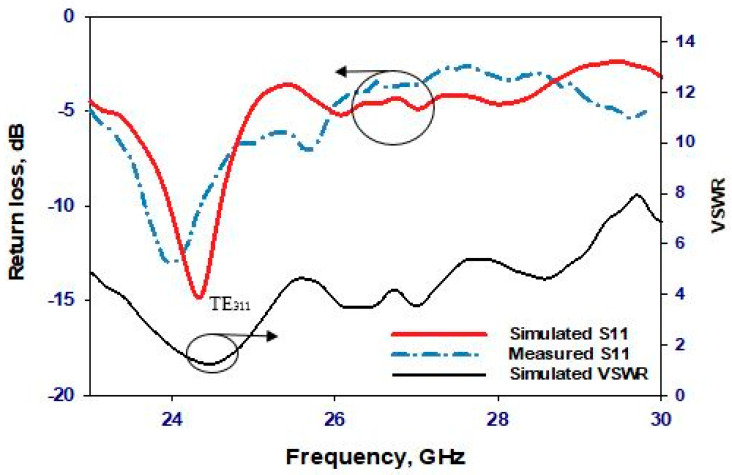
Realized gain when the PIN diode is OFF.

**Figure 12:**
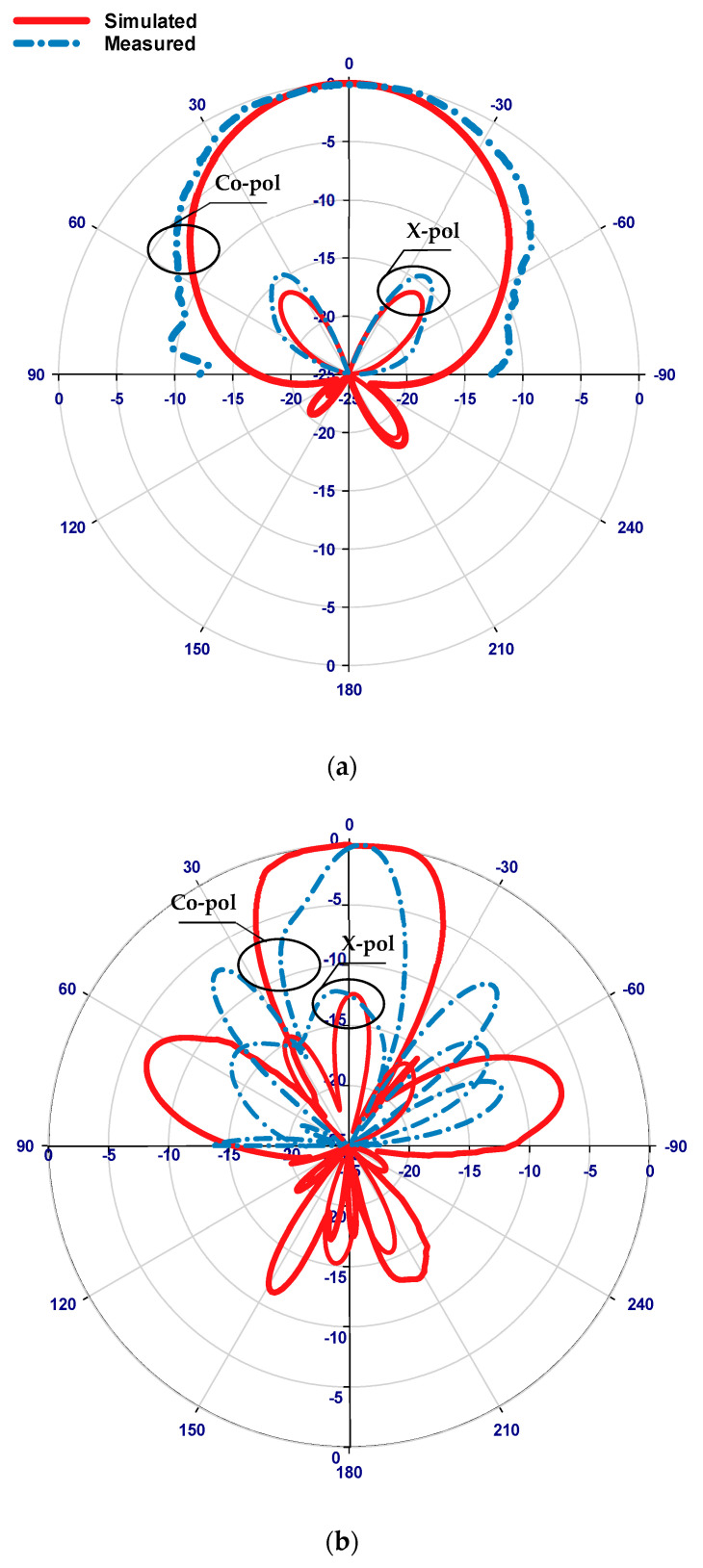
Radiation patterns at 24.3 GHz when the PIN diode is OFF. (**a**) E-Plane, (**b**) H-Plane.

In the first scenario, the PIN diode is set to its reverse-biased OFF state, marking the starting point of our investigation. Figure 10 presents a comparative analysis between simulated and measured reflection coefficients, revealing a commendable level of agreement. The simulated and measured impedance bandwidths stand at 2.3% and 2.66%, respectively, indicating a close alignment between theoretical predictions and experimental observations. Of particular note are the resonance frequencies: while the simulated resonance frequency registers at 24.3 GHz, the measured frequency is slightly lower at 24 GHz, representing a marginal deviation of merely 1.25%. This negligible difference underscores the accuracy of the simulation in predicting the antenna’s behavior under the OFF-state condition. In the same figure (Figure 10), the voltage standing wave ratio (VSWR) is also simulated. The results show that the VSWR is approximately 1.2 at a frequency of 24.3 GHz, indicating minimal signal reflection and efficient power transfer at this frequency. Turning the attention to the broadside realized gains, Figure 11 showcases the simulated gain peaking at 9.8 dBi at 24.3 GHz, whereas the measured gain reaches 9.2 dBi at 24 GHz. These results show that the presence of the parasitic DRA-II enhanced the gain by ~3 dB. Furthermore, Figure 12 provides insight into the radiation patterns at 24.3 GHz, demonstrating a remarkable level of agreement between the simulated and measured data. Although minor disparities exist, likely attributable to inherent measurement errors, the overall consistency between the simulated and measured radiation patterns validates the accuracy of our simulation approach.

### 3.2. ON-State Scenario

**Figure 13:**
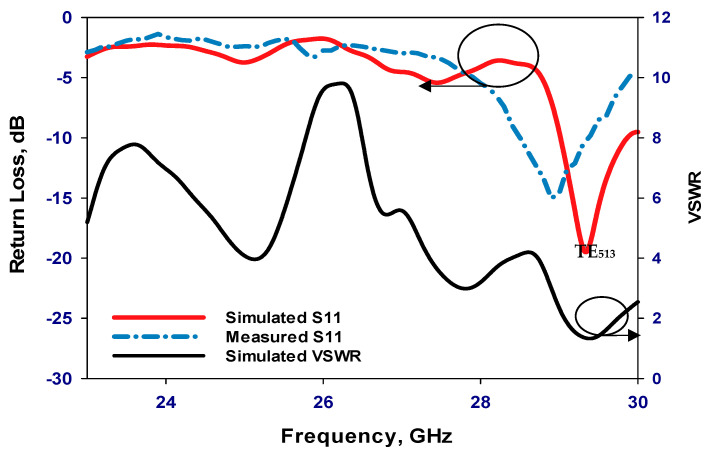
Reflection coefficient and VSWR when the PIN diode is ON.

**Figure 14:**
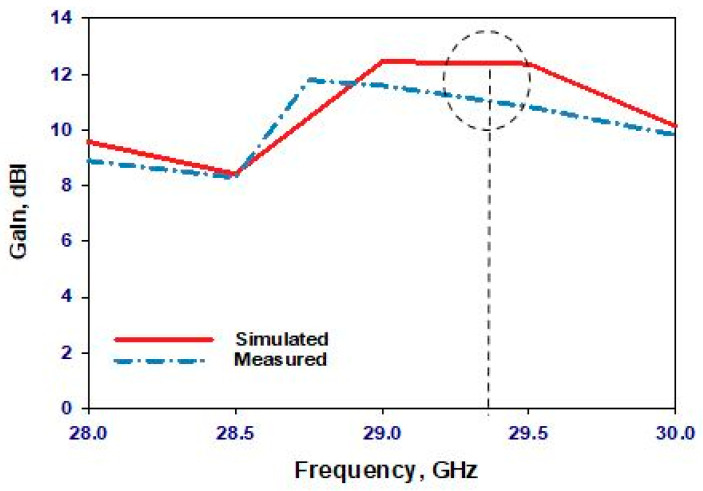
Realized gain when the PIN diode is ON.

**Figure 15:**
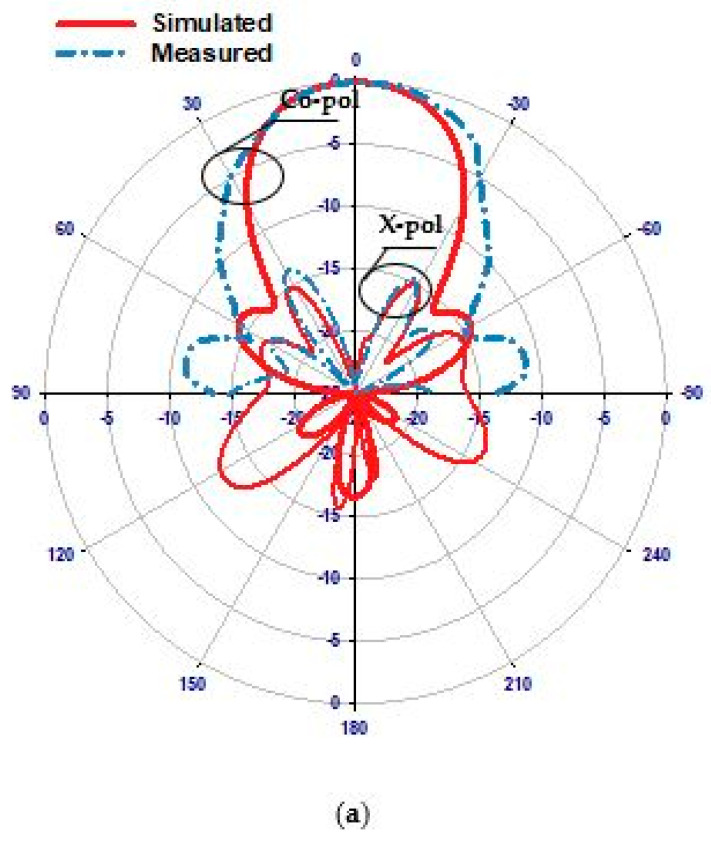

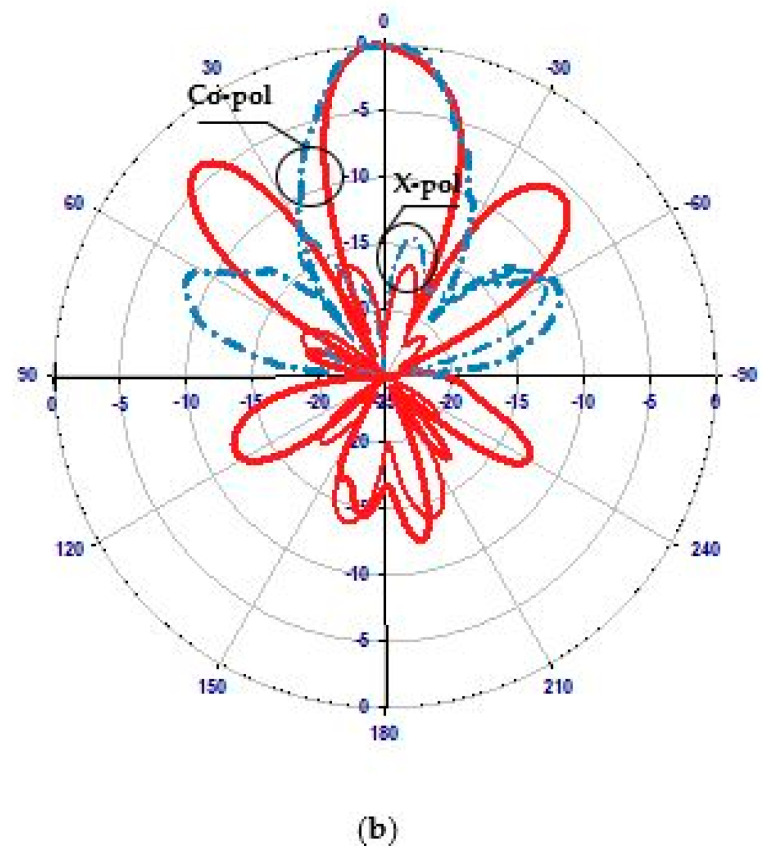
Radiation patterns at 29.3 GHz when the PIN diode is ON. (**a**) E-Plane, (**b**) H-Plane.

Transitioning to the second scenario, the PIN diode undergoes a state change to “ON”. Figure 13 presents a comparative assessment of the simulated and measured reflection coefficients. While the simulated resonance frequency is 29.3 GHz, the measured frequency slightly deviates to 29 GHz, revealing a marginal discrepancy of approximately 1% in the resonance frequencies. Despite this variance, the proximity of these values underscores the reliability of our simulation in capturing the antenna’s behavior under the “ON” state condition. Additionally, attention is drawn to the impedance bandwidths, where Figure 13 highlights respective simulated and measured bandwidths of 2.72% and 2.9%. Although a slight difference exists, this discrepancy remains within an acceptable range, reaffirming the fidelity of our simulation model. Also, Figure 13 illustrates that the VSWR is circa 1.3 at 29.3 GHz, suggesting low signal reflection and efficient power transfer at this frequency. Figure 14 further delves into the comparison between measured and simulated gains, showcasing a notable level of agreement. However, a slight reduction in measured gain to 11.8 dBi at 29 GHz is observed compared to the maximum simulated gain of 12.5 dBi at 29.3 GHz. This discrepancy can be attributed to losses incurred in the biasing network elements, such as the two lumped RF chokes, underscoring the importance of accounting for practical implementation factors in antenna design. The radiation patterns at 29.3 GHz, depicted in Figure 15, exhibit close agreement between simulations and measurements, further validating the accuracy of our simulation approach. While minor deviations may exist, likely due to practical measurement limitations, the overall consistency between the simulated and measured radiation patterns reinforces the credibility of our findings.

**Figure 16:**
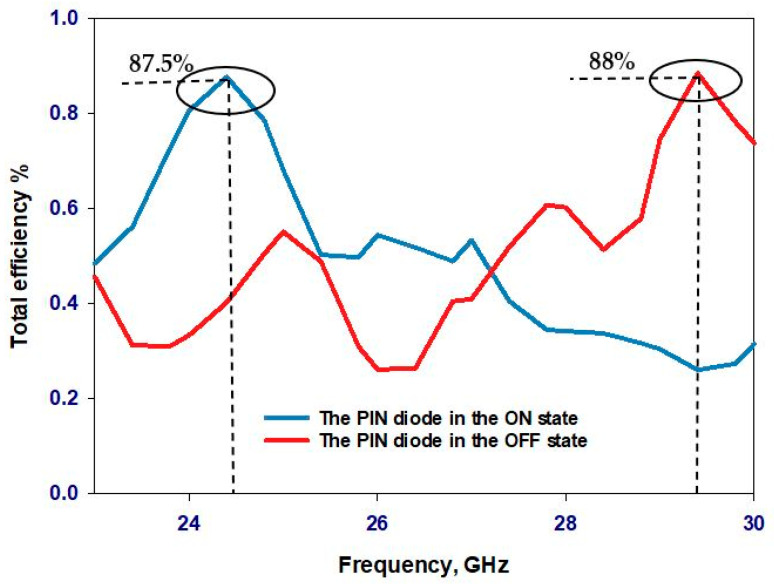
Total efficiency with the PIN diode in ON and OFF states.

**Figure 17:**
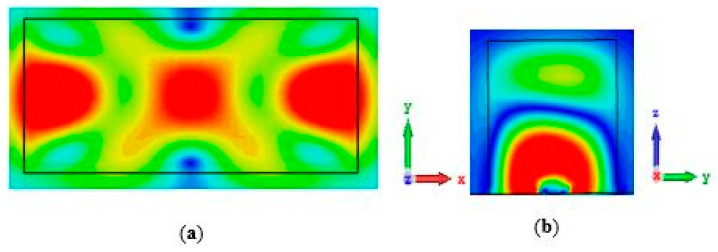
Magnetic fields of the TE_311_ resonance modes at 24.3 GHz. (**a**) *xy* plane; (**b**) *yz* plane.

**Figure 18:**
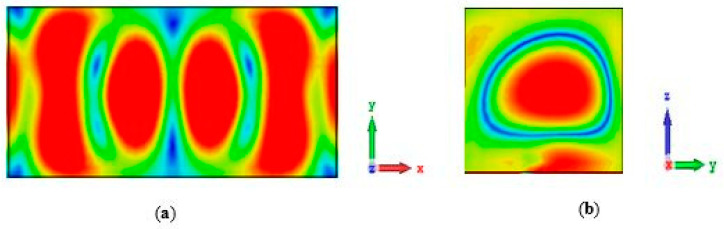
Magnetic field distribution of the TE_513_ resonance modes at 29.3 GHz. (**a**) *xy* plane; (**b**) *yz* plane.

Figure 16 shows the total efficiency of the proposed antenna in both the ON and OFF states of the PIN diode. The simulated total efficiency remains consistent, at approximately 87.5% and 88% at the operating frequencies of 24.3 GHz and 29.3 GHz, respectively. This demonstrates excellent performance for a reconfigurable antenna operating in the mmWave frequency range. The magnetic field distributions within a single DRA are illustrated in Figure 17 and Figure 18, which correspond to the TE_311_ and TE_513_ resonance modes, respectively.

Table 2 compares the performance of the proposed mmWave reconfigurable DRA against the existing counterparts reported in the literature. Notably, literature on practical implementations of mmWave frequency-reconfigurable antennas remains relatively sparse [25], amplifying the significance of our study in filling this gap. The comparison shows that our research marks the pioneering utilization of a DRA as a frequency-reconfigurable mmWave antenna. This distinction underscores the novelty and originality of our research contribution. Additionally, the comparison reveals that our proposed antenna boasts a simpler switching mechanism in contrast to previous works [26,27,28,29,30,31,32]. Furthermore, our antenna achieves a more expansive tuning range compared to references [26,29,30,32], while also delivering superior gain performance relative to the antennas featured in [26,27,30,31,32]. Moreover, our antenna’s achieved frequency-tuning range and gain are on par with those reported in studies employing larger arrays of 16 elements [28]. Conversely, the higher gain reported in [29] can be attributed to using a Fabry–Perot cavity, associated with considerably larger electrical dimensions. Therefore, the proposed antenna offers a simple and compact design while maintaining a wide frequency tuning range and high gain. Its simplicity, compactness, wide tuning range, and high gain collectively position it as a promising candidate for various mmWave communication applications.

## 4. Conclusions

An mmWave antenna has been designed using two rectangular DRA elements and a single PIN diode to achieve frequency reconfigurability through toggling between the ON and OFF states. Only RDRA-I is active at 24.3 GHz when the PIN diode is OFF, providing an impedance bandwidth of 2.66% and a maximum broadside gain of 9.2 dBi. On the other hand, when the PIN diode is ON, both RDRA-I and RDRA-II are excited and operate at 29.3 GHz, offering an impedance bandwidth of 2.9% with a high gain of up to 11.8 dBi. The proposed antenna presents a promising solution for applications that require frequency reconfigurability and high-performance mmWave antennas for 5G and B5G communication systems. The proposed DRA offers a high realized gain in combination with a wide frequency-tuning range and switching simplicity. A wider frequency-tuning range can be achieved by adding more DRAs and additional PIN diodes. In addition, an integrated coplanar strip-line biasing network can further enhance performance.

## Figures and Tables

**Table 1:** Simulated and calculated TE_311_ and TE_513_ modes using CST and MATLAB.

	$\mathrm{Simulated}\;f_{o}$ (GHz)	$\mathrm{Calculated}\;f_{o}$ (GHz)	Resonance Mode
1	24.3	25	TE_311_
2	29.3	30.2	TE_513_

**Table 2:** Performance comparison of mmWave frequency-reconfigurable antenna.

Ref	Antenna	Switch	∆ (%)	Gain (dBi)
[26]	Slot	PIN diode	2.47	6.4
[27]	4-slot array	Photoconductive	30.3	9.0
[28]	16-patch array	Two PIN diodes	24	12
[29]	Fabry–Pérot	Mechanical	8.5	22
[30]	Slot	Mechanical	7	3
[31]	L-Slot	RF MEMS	28	7.9
[32]	Loop	Two PIN diodes	4	7.2
This work	Two DRAs	PIN diode	20	11.8

**∆** is the frequency tuning range.

## Data Availability

Data are contained within the article.
